# Oxidized dextran coated magnetic nanoparticles to develop magnetic cross-linked *Bacillus lehensis* G1 endolevanase aggregates for levan-type fructooligosaccharides synthesis

**DOI:** 10.1371/journal.pone.0333803

**Published:** 2025-10-15

**Authors:** Hotaf Hassan Makki, Nardiah Rizwana Jaafar, Nashriq Jailani, Zaidah Rahmat, Rosli Md. Illias

**Affiliations:** 1 Biology Department, Faculty of Science, University of Tabuk, Umluj, Saudi Arabia; 2 Faculty of Science, Universiti Teknologi Malaysia, Skudai, Johor, Malaysia; 3 Faculty of Chemical and Energy Engineering, Universiti Teknologi Malaysia, Skudai, Johor, Malaysia; Nelson Mandela African Institute of Science and Technology, TANZANIA, UNITED REPUBLIC OF

## Abstract

Endolevanase from *Bacillus lehensis* G1 (rlevblg1) is an enzyme that hydrolyzes levan for the production of levan-type fructooligosaccharides (L-FOS). To maximize the industrial utility of enzymes, it is essential to find effective immobilization techniques that preserve their activity and stability. This study introduced the use of *in silico* analysis to predict the attachment site in order to perform site-directed cross-linking and develop efficient magnetic cross-linked enzyme aggregates (M-CLEAs). According to the computational analysis, dextran with aldehyde groups has attached far from the active site of rlevblg1 compared to dextran with acetyl functional groups. Oxidized dextran (Odex) was prepared and used to coat the magnetic nanoparticles (MNPs). The coated-MNPs (OdexM) exhibited super-paramagnetic behavior (78.9 emu/g), which was applied to develop M-CLEAs of rlevblg1. The biocatalyst (rlevblg1-OdexM-CLEAs) was easily separated from the reaction medium and acquired a recovered activity of 74.7%, maintaining substrate affinity similar to that of the free enzyme. Moreover, rlevblg1-OdexM-CLEAs exhibited great mechanical stability and potential reusability for 10 cycles with more than 50% recovered activity up to the 5^th^ cycle. The optimum temperature of rlevblg1-OdexM-CLEAs has shifted from 30 to 40 °C resulting in a significant improvement in the thermal stability which was 8.9-fold higher than that of free rlevblg1 after 1 h of incubation at 40 °C. After 8 min of incubation at 45 °C, the activity recovery of free rlevblg1 was completely lost, whereas rlevblg1-OdexM-CLEAs retained almost 20% of its initial activity recovery. In addition to the enhanced operational characteristics, M-CLEAs of rlevblg1 hydrolyzed levan into a high yield of L-FOS, proving the suitability of rlevblg1-OdexM-CLEAs as a biocatalyst for industrial production of L-FOS from levan.

## Introduction

The role of enzymes as biocatalysts have been well established in various manufacturing bioprocesses due to their operational, economical and environmental advantages. Enzymes catalyze reactions under mild conditions with fewer operating steps, lower cost, less waste and minimal use of toxic chemicals compared to the typical chemical approach [1,2]. Levansucrase [3], levan fructotransferase (LFTase) [4] and endolevanase [5] are enzymes used for the production of levan-type fructooligosaccharides (L-FOS), which are applied as sugar substitutes without contributing to calories along with being dietary fibers with prebiotic properties that improve gut health and overall well-being [6,7]. These enzymes can be used individually or in combination to optimize the yield and structure of L-FOS.

Regarding to reaction specificity and product profiles, levansucrase catalyzes sucrose, releasing glucose and forming levan that would be partially hydrolyzed to L-FOS [3], whereas LFTase converts levan into cyclic difructose anhydride IV (DFA IV) with a trace of FOS [4]. Moreover, a fusion combining the enzymes’ levansucrase and endolevanase activities was developed to streamline the production process of L-FOS [8,9]. However, this technique presents several issues, including imbalanced enzymatic activities, reduced catalytic efficiency, expression challenges, and limited control over the product profiles, thus leading to the accumulation of levan and an insufficient yield of L-FOS in the final product. On the other hand, endolevanase demonstrates distinct advantages over levansucrase and LFTase in the biosynthesis of L-FOS, particularly in terms of catalytic efficiency, high product purity, and suitability for large-scale industrial applications [10].

Endolevanase secreted by *Bacillus lehensis* G1 (rlevblg1), among other enzymes with potential viability for industrial and commercial purposes [11], is a significant enzyme that belongs to glycoside hydrolases and is classified in the GH32 family [12]. The rlevblg1 operates by cleaving the β (2 → 6) glycosidic bonds in levan for the production of L-FOS [5,12]. Due to its high catalytic activity toward levan and capability to generate L-FOSs with defined degrees of polymerization (DP) ranging from 2 to 10 [7,13], rlevblg1 is a valuable biocatalyst for the development of functional sweeteners in the food industry [14]. However, native enzymes demonstrate poor stability and lack of reusability under harsh industrial conditions, including certain levels of pH and temperature, which hinder the efficient synthesis of the product and increase the production cost [15,16]. Enzyme immobilization is a widely recognized technique that develops operationally stable and reusable biocatalysts, which are advantageous for many industrial bioprocesses [17].

The carrier-free immobilization technique, cross-linked enzyme aggregates (CLEAs) method has attracted increasing attention due to its simplicity in preparation and robustness in industrial applications [18,19]. CLEAs is a practical method that offers several advantages including high catalytic activity, sustainable stability against extreme operating conditions, low production cost due to the exclusion of carriers, ease of synthesis, facile recovery and reusability, as well as the fact that no extensive purification of enzymes is needed [18]. However, formation of CLEAs is challenging for enzymes with a small quantity of external lysine residues [20] that could cause inefficient cross-linking and low mechanical stability of CLEAs, hence leaching of the aggregates and loss of the activity [21]. Moreover, the use of the micromolecular cross-linking agents, such as glutaraldehyde (GA), may lead to the formation of a compact cluster of enzyme aggregates [22] and the interaction with the catalytic amino acids [23], which hinder the internal diffusion of substrate and cause enzyme inactivation [24]. To overcome these limitations, different macromolecular polysaccharide-based cross-linkers have been used to develop CLEAs [25]. However, each cross-linker has a distinct nature and structural characteristics, leading to unique intermolecular interactions, thus varying the level of activity recovery of the formed CLEAs [26].

In our previous study [27], the impact of different macromolecular cross-linkers was investigated to develop efficient CLEAs of rlevblg1 through the combination of computational and experimental analyses. Due to the distanced bonding of dextran from the active site, rlevblg1 cross-linked with dextran (rlevblg1-dex-CLEAs) exhibited the highest binding affinity (−7.1 kcal/mol) and activity recovery compared to six other cross-linkers. The activity recovery of rlevblg1-dex-CLEAs continued to increase and reached 90.5% at the optimum condition with higher thermal tolerance, storage stability, and affinity towards substrates in comparison to the free enzyme. However, the reusability of rlevblg1-dex-CLEAs was limited to five cycles due to enzyme leakage during the separation process. Even though CLEAs generates stable enzymes with a straightforward procedure, separation through centrifugation is costly and challenging to be performed on a large-scale, thus limiting CLEAs application in industrial processes [28].

Fortunately, the separation issue can be overcome via the magnetically recoverable CLEAs, known as magnetic CLEAs (M-CLEAs), where cross-linking is performed in the presence of magnetic nanoparticles (MNPs). M-CLEAs have facilitated the recovery of CLEAs particles from the reaction medium through the adoption of an external field of magnet rather than filtration or centrifugation [29]. Therefore, M-CLEAs generate biocatalysts that are stable with maximized potential of repeated use in continuous processes [30]. After 10 cycles of reuse, glutamyl transpeptidase-M-CLEAs maintained a continuous initial activity (100%) [31] and acylamidase-M-CLEAs retained more than the half (60%) of its initial activity [32].

Nevertheless, the use of native MNPs resulted in leaching of the iron into the medium, especially in acidic environments, making it unsuitable for industrial food related applications [21]. Iron leaching can be prevented by pre-activating the MNPs, followed by subsequently cross-linking the aggregates [33]. However, due to improper activation of the MNPs, the catalytic activity of M-CLEAs can be inhibited by the complicated and undesirable interactions among the MNPs, cross-linker and enzyme molecules, which hinder the access of the substrate into the active site region of the enzyme [34]. In addition, a reduction in the enzymatic recovery could occur due to the weak saturation magnetizability of the MNPs, which makes the magnetic recovery challenging in large-scale processes [28]. These findings justify the need to cover the MNPs and determine the region of the formed linkages to prevent iron leakage and improve substrate accessibility, respectively, in M-CLEAs formation.

**Fig 1:**
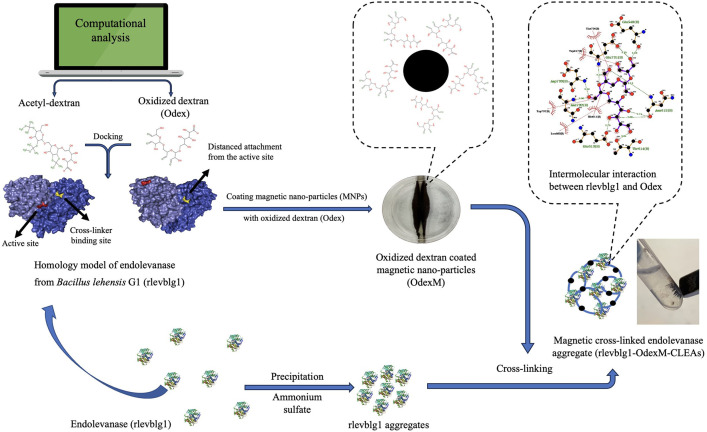
Scheme of the preparation of magnetic cross-linked endolevanase aggregates (rlevblg1-OdexM-CLEAs) with the aid of computational analysis.

Although, dextran was proven to be the optimal cross-linker for rlevblg1, the application of native dextran coated MNPs was withdrawn in some industrial production as a consequence of its weak bonding on the surface of the MNPs [35], hence leaving the bare MNPs in the surrounding environment [36]. In contrast, various studies have reported an enhanced stability of the MNPs when coated with dextran derivatives functionalized with specific chemical groups [37,38]. However, experimentally evaluating the effects of the functionalized dextran derivatives on the activity recovery of the immobilized enzyme is costly and tedious. Furthermore, predicting the binding sites and intermolecular interactions of the cross-linker and MNPs with the enzyme molecules remains a significant challenge in M-CLEA development [34]. Henceforth, the aim of this study is to conduct dextran functionalization based on the findings of computational analysis and then use it to coat the MNPs, thus performing a site-directed cross-linking which guarantees distanced interactions from the active site of the enzyme (Fig 1).

In the current study, modified dextran, with two different functional groups (Acetyl and aldehyde), was docked with the enzyme through *in silico* analysis to identify the best chemical group that can be used to functionalize dextran derivatives prior to coating the MNPs. Due to the distanced attachment from the catalytic region, oxidized dextran was selected as a coating material for the MNPs. The coated-MNPs were used to develop M-CLEAs of rlevblg1, which were then characterized for their optimum pH and temperature, functional group and kinetic analysis, thermal and pH stability, L-FOS production, reusability and mechanical stability. The free rlevblg1 and previous CLEA [39] were used as the primary controls throughout the study. The rlevblg1-M-CLEAs facilitated the recovery of the enzyme, improved thermostability, maximized the reusability and L-FOS production compared to that of the free and CLEA forms of rlevblg1, which confirms the potential of this novel technique to generate promising biocatalysts for L-FOS synthesis from levan.

## Materials and methods

All used reagents and chemicals were of analytical grade and sourced from Thermo Fisher Scientific (USA), Sigma-Aldrich (USA), and Merck (Germany), unless otherwise specified.

### Molecular docking for dextran functionalization

The homology model of endolevanase from *Bacillus lehensis* G1 (rlevblg1) was constructed as described in our previous study [27]. Briefly, the amino acid sequence of rlevblg1 was subjected to similarity search using NCBI–BLAST and HHpred servers to identify suitable structural templates. Fructofuranosidase from *Schwanniomyces occidentalis* (PDB ID: 3KF3) was selected as the template based on its low expectation value (E-value) and >30% sequence identity, which is considered sufficient for generating an accurate model [40,41]. Protein sequences of both the target and template were aligned using ClustalW, and the homology modeling was performed using Modeller 9.13. Among the numerous generated models, the one with the lowest discrete optimized protein energy (DOPE) score and molecular probability density function (PDF) was selected. The final model was evaluated using the root mean square deviation (RMSD) and Ramachandran plot analysis [42].

For suitable functionalization, the.pdb 2D file of dextran acquired from the PubChem database was designed with two different functional groups (acetyl at C_2_-C_3_, C_2_ and C_4_ [43] and aldehyde across C_3_-C_4_ [44]) using the ChemSketch program. Based on the SMILES (https://cactus.nci.nih.gov/translate/), the acquired mol documents were transformed into pdp documents. The formerly established 3D rlevblg1 homology model [27] was docked with acetyl-dextran and aldehyde-dextran (pdp formats) through the AutoDock Vina (1.5.6, 2014) software [45]. Meanwhile, this study demonstrated the PyMOL [46] utilization for all graphical representations of the 3D models. The 2D cross-linker (ligands) non-covalent interaction diagrams with the residues on the surface of rlevblg1 were pictured based on the LigPlus program (version 2.2.4) [47]. Moreover, the functionalization of dextran derivatives was established, corresponding to the non-covalent pre-association regions interactions between the receptor and ligands, which would predict the site of the subsequent covalent bond formation observed experimentally [40,48,49].

### Dextran oxidation

Oxidation of dextran was performed as described by Muangsiri and Kirsch [50] with some modifications. Briefly, 10 g of dextran was dissolved in 800 mL of deionized water (dH_2_O). Subsequently, varying quantities of sodium periodate (0.25, 0.5, 0.75, and 1 M) were mixed with 200 mL dH_2_O. The resulting solutions were continuously stirred with the dextran solution. Furthermore, the reactions occurred without light at room temperature for different reaction periods (0.5, 1, 2, and 4 h). The oxidizing process was halted by combining 95% ethanol with the reaction solution, precipitating the oxidized dextran (Odex). The powder form of the solution was then produced by precipitating Odex through (95%) ethanol washing and filtration. Lastly, the resulting Odex was placed to dry in the oven overnight at 50 °C and kept in the dark for further utilization. The highest aldehyde content determined the best value for each parameter.

### Aldehyde content determination

The aldehyde group amount generated during the reaction between sodium periodate and dextran was calculated using a modified hydroxylamine hydrochloride method [50]. Approximately 0.1 g of oxidized dextran (Odex) was mixed with hydroxylamine hydrochloride (0.25 M, 25 mL). Subsequently, the solution was adjusted to pH 5 with 5 M sodium hydroxide (NaOH) and incubated at 50 °C in a water bath for 2 h at 100 rpm. The reaction mixture was titrated rapidly with standardized 5 M NaOH to the endpoint of pH 5. The V_control_ (mL) of native dextran was utilized as a control to measure the consumption of NaOH. Afterwards, the aldehyde content was determined using equation (1) by observing the NaOH consumption of V_sample_ (mL) at pH 5 as follows:


$$\text{Aldehyde}\;\text{content}\;\;(\%)=\frac{\;V_{sample\;}-\;V_{blank}\;\times \;M\;\times \;\text{MW}\;}{m\;\times \;\text{1000}}\times \;\text{100}\%$$ (1)

where V is the NaOH titrating amount and M is the NaOH concentration. The MW stands for the average molecular weight of the dextran repeating unit and m is the dextran mass used for quantification. In this study, M was 0.1 mol L^-1^, MW was 504.438 g mol^-1^, and m was 0.1 g.

### Magnetic nanoparticles (MNPs) preparation

Magnetic nanoparticles (MNPs) were prepared as previously described by Khalil [51] with minor modifications. Anhydrous ferric chloride (FeCl_3_, 6.48 g) was thoroughly dissolved in dH_2_O (150 mL) to prepare aqueous solution A. On the other hand, aqueous solution B was produced by dissolving 6.584 g of potassium iodide (KI) in dH_2_O (50 mL). The two solutions were combined at room temperature and agitated at 600 rpm for 1 h to attain equilibrium. Subsequently, the iodine component was filtered and washed with dH_2_O. The 25% NaOH was added dropwise while stirring continuously (600 rpm) to the entire filtrate volume for hydrolysis. This process was performed until the black MNPs were completely precipitated and the pH of the mixture was between pH 9–11. Lastly, the mixture was centrifuged at 3000 × g for 10 min at 27 °C; the MNPs were washed with dH_2_O until the pH was adjusted to pH 7 and dried at 70 °C.

### Coating of the MNPs

Coating of the MNPs with Odex was performed as previously described by Predescu et al. [52]. Various concentrations of dried MNPs (0.025, 0.05, 0.1, 0.2, and 0.4% (w/v)) were added to 1% (v/v) of the solution of Odex and placed in a water bath at 100 °C for 1 h. After the solution was cooled to room temperature, centrifugation of the mixture was performed at 800 × g for 15 min to separate the newly produced components. The finished product of coated MNPs with Odex (OdexM) was magnetically separated, washed with methanol, and left to dry for overnight at room temperature.

### Construction of rlevblg1-OdexM-CLEAs

Expression and purification of rlevblg1 were conducted as described in our previous reports [27,39]. Moreover, M-CLEAs of rlevblg1 was prepared, using the optimal condition of rlevblg1-dex-CLEAs [39], by the addition of 60% (v/v) ammonium sulfate into 0.6 mg/mL of the enzyme with continued agitation (200 rpm) for 1 h at 4 °C. Next, the precipitated rlevblg1 was cross-linked with 1% (w/v) of OdexM, using different Odex (mg/mL) to MNPs (mg/mL) (1:0.025, 1:0.05, 1:0.1, 1:0.2, and 1:0.4) ratios in 1 mL total volume with a buffer of pH 10 (50 mM glycine-NaOH buffer). The cross-linking step was performed at 4 °C for 2 h under 200 rpm of shaking. The formed rlevblg1-OdexM-CLEAs was then separated using an external magnetic field at room temperature, washed with glycine-NaOH buffer (50 mM, pH 8), and stored in the same buffer at 4 °C for further analysis. The rlevblg1-OdexM-CLEAs with the highest activity recovery, calculated from equation (2), determined the optimal concentration of OdexM that can be used to form M-CLEAs.


$$\;\;\;\;\;\;\;\;\;\;\;\;\;\;\;\;\;\;\;\;\;\text{Recovery}\;\text{activity}\;(\%)=\frac{\text{Activity}\;\text{of}\;\text{M}-\text{CLEAs}\;\left(\frac{\text{U}}{\text{mg}}\right)}{\text{Activity}\;\text{of}\;\text{free}\;\text{enzyme}\;\left(\frac{\text{U}}{\text{mg}}\right)}\;\times \text{100}\;\%$$ (2)

where the enzyme activity was calculated by dividing (sugar released (μmol) × total volume of assay (mL)) by (volume of enzyme (mL) × time of assay (min) × volume in cuvette (mL)).

### TEM and VSM analyses

The MNPs and OdexM morphologies were evaluated by transmission electron microscopy (TEM), utilizing a JEOL 2000 FX instrument, to investigate the efficacy of the oxidized dextran polysaccharide coating on the MNPs surface. Using a vibrating sample magnetometer (VSM), the magnetic characteristics of MNPs and OdexM were evaluated. The samples were magnetized by having them situated in a consistent magnetic field and then mechanically vibrated sinusoidally with a piezoelectric material. The hysteresis plot of the samples was obtained by field calculation of an external electromagnet. Furthermore, the samples underwent a concentration process under vacuum with an Eppendorf Concentrator Plus^TM^ (Eppendorf AG, Hamburg, Germany) to eliminate additional H_2_O, before TEM and VSM.

### FT-IR, FESEM and particle size analyses

The surface morphology of Odex, MNPs, OdexM, and rlevblg1-OdexM-CLEAs was investigated using a field emission scanning electron microscope (FESEM), (Hitachi SU8020, USA). FESEM images were taken at 3,000 × magnification. Meanwhile, the Fourier Transform-infrared (FT-IR) spectroscopy (Perkin Elmer, Ohio, USA) (500–4000^−1^) determined the functional components of Odex, MNPs, OdexM, and rlevblg1-OdexM-CLEAs. The materials underwent vacuum concentration to eliminate additional H_2_O through the Eppendorf Concentrator Plus^TM^ (Eppendorf AG, Hamburg, Germany) before being subjected to FESEM and FT-IR. In addition, the dynamic light scattering (DLS) analysis via the Malvern Zetasizer Nano ZSP was used to measure the particle sizes of Odex, MNPs, OdexM, and rlevblg1-OdexM-CLEAs (Malvern Panalytical, Malvern, UK).

### Operational characterization

#### Optimum temperature and pH.

The optimal temperature for rlevblg1-OdexM-CLEAs was determined by testing temperatures from 20 °C to 50 °C over 10 min. Similarly, the optimal pH for M-CLEAs of rlevblg1 was assessed across pH values from 6 to 11 at 30 °C for 10 min. The experimental buffers used included 50 mM sodium acetate (pH 5.0), 50 mM potassium phosphate (pH 6.0–7.0), and 50 mM glycine-NaOH (pH 8.0–11.0) [53]. The activity levels at different temperatures and pH values were calculated relative to the optimal condition, which was set at 100%.

#### Thermal and pH stability.

The thermal stability of free and M-CLEAs of rlevblg1 was assessed by measuring their residual activity after being incubated in a 50 mM glycine-NaOH buffer (pH 8.0) without a substrate (levan) at 35 and 40 °C for 1 h, meanwhile, for the duration of 12 min at 45 °C. The enzymatic activity was recorded at intervals of 10 min at 35 and 40 °C, while it was observed every 2 min at 45 °C. Residual rlevblg1 activity was then determined through enzyme assays and expressed as a percentage of the initial activity, which was considered 100%.

For pH stability, the rlevblg1-OdexM-CLEAs was pre-incubated without a substrate (levan) in different pH buffers ranging from pH 6–11 at 4 °C for 1 h. The stability across pH levels was evaluated using enzyme assays, with the relative activity calculated against the optimal condition set at 100%.

#### Kinetic and effectiveness factor analyses.

At various levan concentrations (1.0 to 25.0% (w/v)), the kinetic characteristics of rlevblg1-OdexM-CLEAs were measured, including maximum velocity (V_max_), Michaelis-Menten constant (*K*_*m*_), turnover number (k_cat_), and catalytic efficiency (k_cat_/*K*_*m*_). The V_max_ and *K*_*m*_ computations utilized a Lineweaver-Burk plot of 1/v versus 1/s. In addition, the same concentrations of levan (1.0 to 25.0% (w/v)) were used to test the initial reaction rates and calculate the effectiveness factor (η) of rlevblg1-OdexM-CLEAs. Equation (3) measures η for M-CLEAs as follows:


$$(\eta )=\frac{V_{M-CLEAs}\;}{V_{free\;enzyme}\;}$$ (3)

#### Mechanical stability and leaching-out analyses.

Mechanical stability and leaching analyses of rlevblg1-OdexM-CLEAs were performed as previously reported by Abd Rahman et al. [53]. The M-CLEAs of rlevblg1 was subjected to a continuous stirring under different agitation speeds (100, 150, 200, and 250 rpm), at 30 °C, in 2 mL of glycine-NaOH buffer (50 mM, pH 8). After 30 min of agitation, rlevblg1-OdexM-CLEAs was recovered by the use of an external magnetic field and the catalytic activity in the supernatant was measured via enzyme assay to determine the amount of enzyme leakage. The leaching out percentage of rlevblg1 was calculated using equation (4) as follows:


$$\text{Leached}\;\text{out}\;(\%)=\frac{\text{rlevblg1}\;\text{activity}\;\text{in}\;\text{supernatant}\left(\frac{\text{U}}{\text{mg}}\right)\;\;}{\text{Initial}\;\text{activity}\;\text{of}\;\text{immobilized}\;\text{rlevblg1}\;\left(\frac{\text{U}}{\text{mg}}\right)}\;\times \text{1}\text{0}\text{0}\%\;$$ (4)

#### Reusability.

The recyclability attribute of M-CLEAs was successfully evaluated for ten cycles. Each cycle finished with the immobilized rlevblg1 being recovered using an external magnetic field, cleaned in a glycine-NaOH buffer (50 mM, pH 8), and undergoing resuspension in a new reaction solution. Under conventional enzyme assay conditions, the activities of M-CLEAs were assessed. At 100%, the enzyme activity from the initial cycle was measured. After every subsequent cycle, the remaining enzyme activity was computed.

### L-FOS production

The stability and kinetics of levan-type fructooligosaccharides (L-FOS) conversion using M-CLEAs of rlevblg1 were examined following the method established by Abd Rahman et al. [5]. A 0.5% levan solution (1100–1600 kDa) was added to rlevblg1-OdexM-CLEAs, which were then incubated in a thermomixer (Eppendorf, Hamburg, Germany) for 1, 2, and 3 h at 30 °C while stirring at 850 rpm. The conversion of levan to L-FOS and reducing sugars was analyzed using high-performance liquid chromatography (HPLC) with a refractive index detector (Waters, Milford, MA, USA). For separation, a Cosmosil Sugar-D column (4.6 mm × 250 mm) was utilized, kept at a temperature of 30 °C. The detector sensitivity and internal temperature were adjusted to 64 MV and 37 °C, respectively. The mobile phase consisted of a 70:30 acetonitrile–water mixture, flowing at a rate of 1.0 mL/min. The concentrations of monosaccharides and oligosaccharides were calculated using an equation derived from a standard calibration curve (S1-S6 Figs). Standards included a mixture of monosaccharides (fructose) and oligosaccharides (levanbiose; DP2, levantriose; DP3, levantetraose; DP4, and levanpentaose; DP5), sourced from Sigma-Aldrich with a HPLC purity of 99%. All standards and samples underwent centrifugation at 10,652 × g for 15 min at 25 °C and were filtered through a 0.22 μm syringe filter before injecting 20 μL samples into the column. The extent of bioconversion was determined by measuring the substrate consumption.

### Statistical analysis

Statistical analyses for dextran oxidation and M-CLEAs characterization were conducted using analysis of variance (ANOVA) and Tukey’s multiple comparison post-test with GraphPad Prism 5.0 (GraphPad Software, Inc., San Diego, CA). Differences between the analysis groups were considered not significant (indicated by “ns”) at a P value greater than 0.05. All tests were performed in triplicates with a significance level of 95%.

## Results and discussion

### Molecular docking analysis

Coating of the magnetic nanoparticles (MNPs) is substantial, especially in food related industrial productions, owing to their cytotoxicity. Even though native dextran is the optimal cross-linker for endolevanase from *Bacillus lehensis* G1 (rlevblg1), as a coating material, it forms weak interactions with the surface of the MNPs that could easily break and expose iron oxide to the reaction mixture [35,36]. On the other hand, several studies discussed the improvement in the stability of coated-MNPs with functionalized dextran with different functional groups [37,38]. However, screening the effect of designing dextran derivatives with multiple functional groups on the activity recovery consumes time, effort and material. Additionally, predicting the binding site and intermolecular interaction to avoid complex interactions with the enzyme molecules is challenging [34].

**Fig 2:**
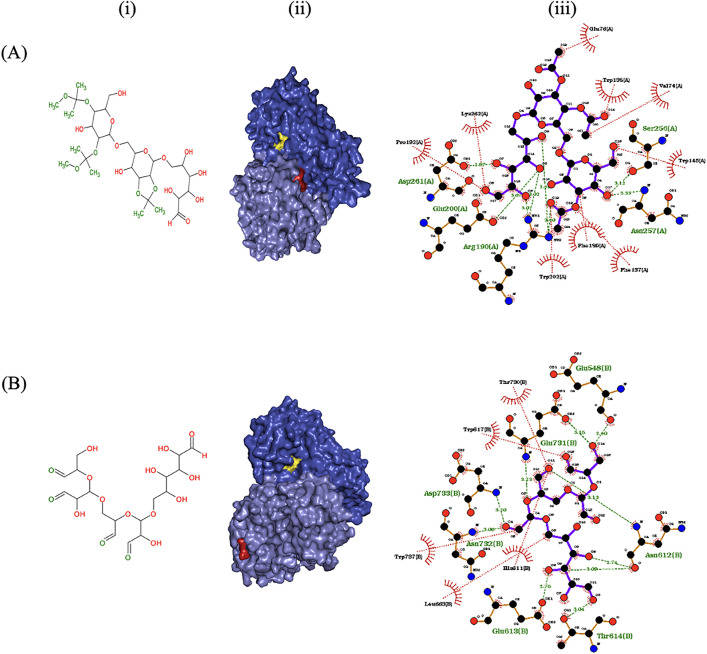
Docking analysis between rlevblg1 and modified dextran with two different functional groups. (A) acetyl and (B) aldehyde. (i) 2D structure of the modified dextran generated by ChemSketch, where functionalization sites are represented in green, (ii) 3D schematic diagram of the cross-linker interacting with the homology model of rlevblg1 through the AutoDock Vina software, where the catalytic site appears in yellow and cross-linker binding site is in red, and (iii) 2D schematic diagram interaction devaloped by LigPlus program, where the purple line represents the ligand, green dashed line represents hydrogen bond, and red dashed line represents hydrophobic interaction.

Herein, docking simulations were utilized to predict the non-covalent bindings between the ligands and specific amino acid residues of the previously constructed 3D structure of rlevblg1 [27], which served as the receptor molecule. These interactions identify the potential attachment sites of the covalent cross-linking, which would be located in close proximity within the predicted non-covalent regions [40,48,49]. Accordingly, the modification of dextran derivatives with two different functional groups (acetyl at C_2_-C_3_, C_2_ & C_4_ [43] and aldehyde at C_3_-C_4_ [44]) was performed using the ChemSketch program (Fig 2). The 3D structures of the modified dextran were used as ligands to generate the conformation of ligand-rlevblg1 complexes and determined their free binding energy values. Based on the computational analysis, the binding affinity of rlevblg1 interacting with acetyl-dextran (−6.3) was lower than that of aldehyde-dextran (−5.8). The protein-ligand complex with lower binding energy demonstrates better affinity and stability compared to that of higher binding energy [54,55].

Further analyses of the non-covalent binding sites and amino acids involved in the intermolecular interactions were performed to pre-determine the regions of covalent linkage during cross-linking reactions [40,48,49]. These analyses were investigated to prevent any interference of the cross-linker with the active site, causing substrate diffusion problem and enzyme inactivation [56]. Acetyl-dextran interacted with amino acids that were close to the catalytic site of rlevblg1, indicating possible enzyme deactivation and substrate inaccessibility. On the other hand, aldehyde-dextran interacted with different amino acids. As shown in Fig 2, aldehyde-dextran established interactions associated with Asp733, Asn732, Thr614 Asn612, Glu731, Thr730, Trp737, Leu663 and His611 of rlevblg1, which were also involved in the interactions of native dextran (the optimal cross-linker for rlevblg1) [27]. Importantly, all of these interactions were distanced from the catalytic region, thus substrate accessibility and diffusion would not be affected or interfered with. Therefore, aldehyde-dextran was chosen to act as a coating material for MNPs due to the role of the attachment site of the cross-linker on the enzyme kinetic and performance after immobilization [57].

### Dextran oxidation

Dextran oxidation with sodium periodate is a commonly used method to introduce aldehyde groups onto dextran molecules [58]. In a mild oxidation process, sodium periodate reacts with the vicinal diol functional groups of dextran polymer and breaks the C-C bond between C_2_-C_3_ or C_3_-C_4_ to form two aldehyde groups [59]. However, the cleavage of C_3_-C_4_ is more dominant during dextran oxidation [44]. On the other hand, double oxidation may occur on account of the second attack to the vicinal hydroxyl group in the aldehyde at C_3_, resulting in the separation of C_3_ from dextran molecules as a side product (formic acid) [60,61]. Therefore, the influence of sodium periodate concentration and reaction time was investigated to determine the optimal reaction condition for dextran oxidation. The level of oxidation was measured using the hydroxylamine hydrochloride method at which the amount of aldehyde groups represents the number of carbonyl groups per 100 glucose units of dextran [62].

**Fig 3:**
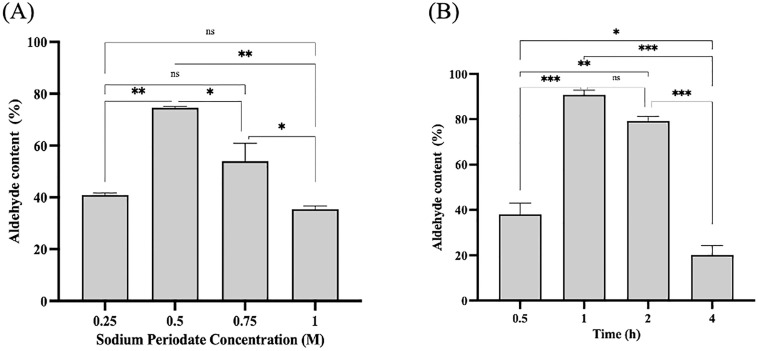
Optimization of oxidized dextran (Odex) formation. Effect of (A) sodium periodate concentration and (B) reaction time. The experiments were performed in triplicate, and error bars indicate standard deviations. Average values that show significant differences are highlighted with varying numbers of asterisks. This is based on the results from a one-way ANOVA followed by Tukey’s multiple comparisons test, using a 95% confidence interval: p < 0.05 (*) indicates significance; p < 0.01 (**) indicates a more significant difference; p < 0.001 (***) denotes high significance, while (ns) signifies a non-significant result.

Based on Fig 3 (A), the highest degree of oxidation and maximum aldehyde content on the surface of dextran (74.6%) was observed as 0.5 M of sodium periodate was applied. However, the aldehyde content gradually decreased with a higher concentration of sodium periodate. The reduction in the oxidation level is attributed to the initiation of double oxidation process as the increase of sodium periodate concentration would lead to further oxidation for formic acid formation, resulting in the decline of aldehyde content [60,63]. After the proper periodate concentration (0.5 M) was determined, the effective oxidation time was investigated. The highest aldehyde content was (90.7%) at 1 h of reaction and decreased to 20.1% at 4 h of reaction (Fig 3 (B)). Short oxidation time caused insufficient oxidation, whereas long reaction time led to the formation of tightly bound molecules [58], hence hindering the oxidation process. As a result, 1 h was chosen as the optimal reaction time.

### Coating of magnetic nanoparticles (MNPs) and development of rlevblg1-OdexM-CLEAs

The oxidized dextran (Odex) was used to coat the formed MNPs and develop oxidized dextran coated MNPs (OdexM), which maintained superior magnetic activity and was easily recovered using an external magnetic field. However, the best coating ratio was determined based on the retained activity recovery of the immobilized rlevblg1. Therefore, the formation of M-CLEAs was performed using the optimized condition of rlevblg1-dex-CLEAs [39]. However, the aggregated enzyme was cross-linked with 1% (w/v) of OdexM that was prepared with different ratios of Odex (mg/mL) to MNPs (mg/mL) (1:0.025, 1:0.05, 1:0.1, 1:0.2, and 1:0.4).

**Fig 4:**
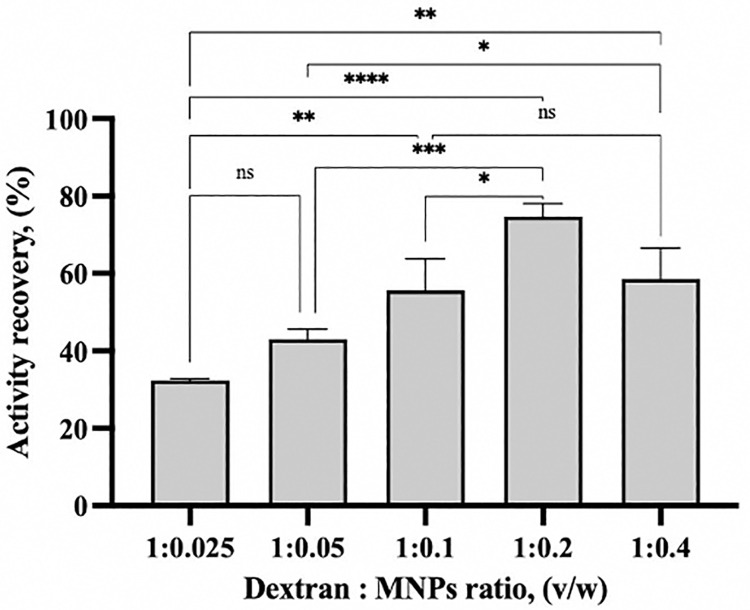
The influence of the cross-linker concentration on the activity recovery of rlevblg1-OdexM-CLEAs. The experiments were performed in triplicates, error bars indicating standard deviations. Average values that show significant differences are highlighted with varying numbers of asterisks. This is based on the results from a one-way ANOVA followed by Tukey’s multiple comparisons test, using a 95% confidence interval: p < 0.05 (*) indicates significance; p < 0.01 (**) indicates a more significant difference; p < 0.001 (***) denotes high significance; p < 0.0001 (****) indicates an extremely significant difference, while (ns) signifies a non-significant result.

Based on Fig 4, the highest activity recovery (74.7%) was obtained using 1% (w/v) of the ratio of 1 mg/mL Odex: 0.2 mg/mL MNPs to cross-link rlevblg1. However, the activity recovery has decreased using below or above the optimal ratio due to insufficient or excessive cross-linking [64], respectively. Low concentration of the cross-linker forms unstable CLEAs leading to enzyme leaching into the reaction mixture and causing a reduction in the activity recovery [65]. On the other hand, at a high concentration of the cross-linker, multi-interactions are formed which could oversaturate and distort the enzyme as well as block the active site and limit substrate accessibility, resulting in decreasing the activity recovery [37]. Thus, 1:0.2 of Odex (mg/mL) to MNPs (mg/mL) is the optimal ratio to develop OdexM and form efficient M-CLEAs of rlevblg1.

### Characterization of OdexM

**Fig 5:**
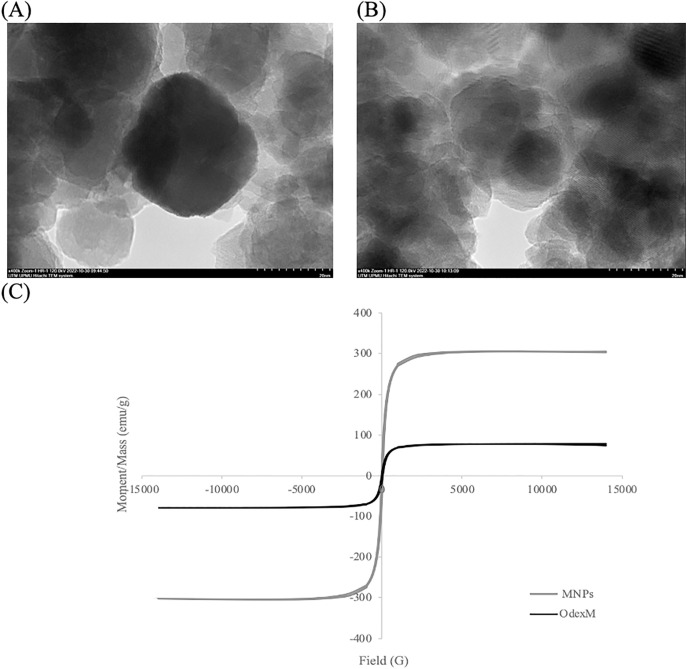
TEM and VSM analyses. TEM images of (A) bare MNPs and (B) MNPs coated with oxidized dextran (OdexM). (C) Magnetization curves of bare MNPs and OdexM using vibrating sample magnetometer (VSM).

The quality of surface coating and magnetic properties of OdexM were observed using TEM and VSM analyses, respectively, then were compared to bare MNPs. Based on TEM images (Fig 5 (A&B)), both samples have spherical and mostly monodispersed shapes. However, MNPs appeared as dark regions due to their high electron density [52], whereas coated MNPs (OdexM) displayed clear crystalline nanostructures, confirming successful coating of the particles. Furthermore, according to the VSM analysis in Fig 5 (C), both samples exhibited a superparamagnetic nature at 300 K, which was confirmed with the absence of the hysteresis [66,67]. However, there was a reduction in the saturation magnetization value of OdexM due to the presence of the non-magnetic material [66] (oxidized dextran). Coating of MNPs minimizes the magnetic strength and stimulates inert behavior to the final nanostructure [68]. Despite the coverage effect, the value of saturation magnetization of OdexM was found at 78 emu/g, which is sufficient for magnetic separation with an external magnetic field. Since the magnetic strength is particularly measured by the saturation magnetization value [67], OdexM holds a superparamagnetic property that would facilitate the recovery of the aggregates using an external magnetic force, thus reducing the risk of enzyme leaching into the reaction environment.

### Morphological changes and particle size distribution

**Fig 6:**
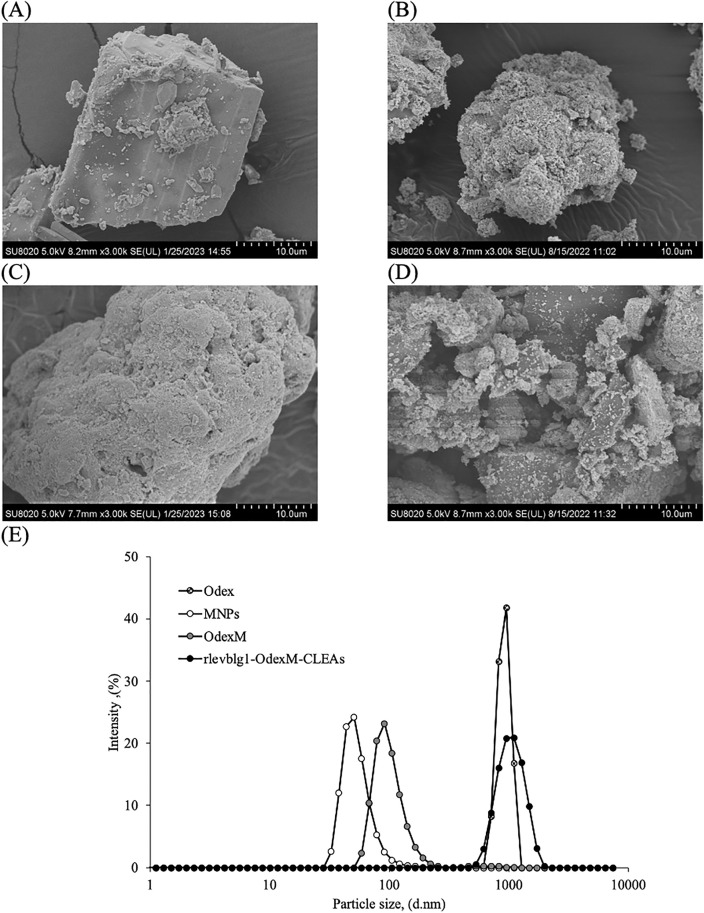
Surface morphology and particle size distribution. FESEM images of (A) oxidized dextran (Odex), (B) bare magnetic nanoparticles (MNPs), (C) oxidized-dextran-coated MNPs (OdexM), and (D) rlevblg1-OdexM-CLEAs at 3,000 × magnification. (E) The particle size distribution using Dynamic light scattering (DLS).

Surface morphology of Odex, MNPs, OdexM, and M-CLEAs of rlevblg1 was investigated by FESEM images at 3,000 × magnification (Fig 6 (A-D)). The structural changes in Odex were compared with the morphology of native dextran [39]. Based on Fig 6 (A), oxidized dextran (Odex) appeared with a rougher surface compared to native dextran, which is consistent with the study conducted by Rai et al. [69]. Moreover, in comparison to bare MNPs (Fig 6 (B)), oxidized dextran coated MNPs (OdexM) (Fig 6 (C)) exhibited larger shapes with rougher surfaces owing to the formation of a compact composition and the existence of biopolymer shells [70]. FESEM images of rlevblg1-OdexM-CLEAs (Fig 6 (D)) showed that M-CLEAs had more porous and spherical structures with rougher surface than that of rlevblg1-dex-CLEAs [39].

In addition, the morphology alteration of the samples was confirmed by the changes in the particle size (Fig 6 (E)). Odex exhibited 100-fold higher particle size distribution compared to native dextran [39]. The difference in surface morphology and particle size is attributed to the influence of sodium periodate that changes the structural integrity of the polysaccharides, thus affecting their particle size after the oxidation process [53]. Furthermore, the average particle size distribution of OdexM was 2-fold higher than that of uncoated MNPs due to the layer of oxidized dextran attached to the surface of MNPs [37,66]. In accordance with these results, a successful coating was confirmed, which would ensure the occurrence of site directed and efficient cross-linking process of M-CLEAs. Moreover, the particle size of rlevblg1-OdexM-CLEAs was 10-fold larger than that of rlevblg1-dex-CLEAs [39], which is in compliance with the lower *K*_*m*_ value and higher effectiveness factor obtained in rlevblg1-OdexM-CLEAs. These results are consistent with the findings of Ifko et al. [28], where M-CLEAs of cellulase had a similar morphology but larger particle size than that of CLEAs as a result of the addition of the MNPs.

### Functional group analysis

**Fig 7:**
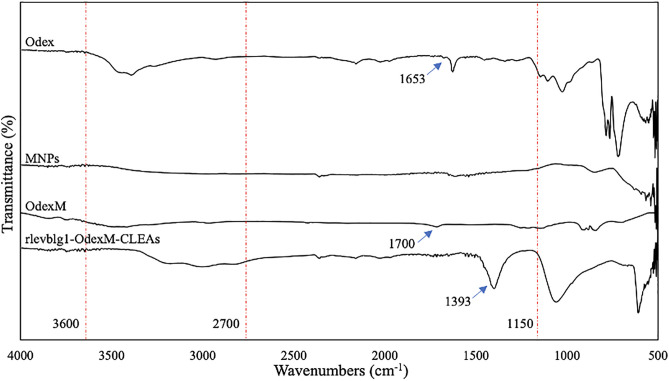
Fourier Transform-infrared (FT-IR) analysis. FT-IR of oxidized dextran (Odex), magnetic nanoparticles (MNPs), oxidized-dextran-coated MNPs (OdexM) and M-CLEA of rlevblg1.

Characterization of the chemical structure and functional group compositions of Odex, MNPs, OdexM, and rlevblg1-OdexM-CLEAs were investigated using FT-IR. Based on Fig 7, compared to the spectrum of native dextran [39], there is a change in the intensity of the characteristic peaks at the regions 3600−3200 cm^−1^ and 1150–1085 cm^−1^ associated to the O–H and C–O–C stretching vibrations [66], respectively, which could be due to the conversion into C–H–O group after the oxidation process. Moreover, oxidized dextran displayed a decrease in the intensity of peak 2920 cm^−1^ correlated to C–H stretching vibrations [63,66]. The oxidation of dextran was confirmed by the increase in the intensity of the peak near 1630 cm^−1^ that corresponded to the C = O stretching vibration [63], and the presence of the new shoulder peak at 1653 cm^−1^ [53] due to the introduction of carbonyl groups, thus indicating the successful functionalization of dextran.

FT-IR spectrum of uncoated MNPs demonstrated Fe–O bands in the area 580−540 cm^−1^ [34], whereas the coating of MNPs and formation of OdexM was confirmed by the broad peak in the area 3600–3200 cm^−1^ corresponding to the O–H stretching of the polysaccharide [63]. In addition, the presence of peak 2920 cm^−1^ assigned to C–H stretching vibrations in OdexM [63,66], which was absent in the spectra of uncoated MNPs. Coating with oxidized dextran was also determined via the peak at 1700 cm^−1^ assigned to C = O bend of the aldehyde group [71] and the adsorption bands in the region 1150−1085 cm^−1^, which corresponds to the stretching of C–O–C [66]. The results of FT-IR spectrum indicate a successful coating of the MNPs.

The spectrum of rlevblg1-OdexM-CLEAs demonstrated a broad peak at the region 3300–2700 cm^−1^ assigned to the O–H stretching vibration [72]. The peak near 1060 cm^−1^ is associated with the C–O stretching vibration of C–O–C linkages [73], whereas the peak close to 1393 cm^−1^ is corresponded to the C–N bend of amide III [74]. Compared to the spectrum of rlevblg1-dex-CLEAs [39], M-CLEAs of rlevblg1 displayed a more intense peak at 1393 cm^−1^ indicating the formation of more amide interactions between the enzyme and OdexM.

### Operational stability of rlevblg1-OdexM-CLEAs

#### Optimum pH and pH stability.

**Fig 8:**
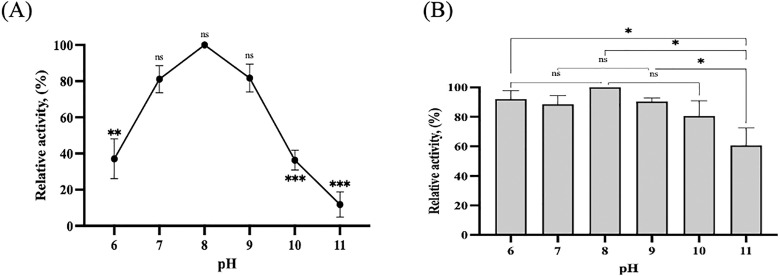
pH optimum and pH stability. (A) The pH optimum of rlevblg1-OdexM-CLEAs. (B) The pH stability of rlevblg1-OdexM-CLEAs. The highest activity of rlevblg1-OdexM-CLEAs was defined as 100% of relative activity. The experiments were performed in triplicate, and error bars indicate standard deviations. Average values that show significant differences are highlighted with varying numbers of asterisks. This is based on the results from a one-way ANOVA followed by Tukey’s multiple comparisons test, using a 95% confidence interval: p < 0.05 (*) indicates significance; p < 0.01 (**) indicates a more significant difference; p < 0.001 (***) denotes high significance, while (ns) signifies a non-significant result.

The optimum pH of rlevblg1-OdexM-CLEAs was determined by performing the enzyme activity assay in solutions with different pH values ranging from pH 6–11 (Fig 8 (A)). M-CLEAs of rlevblg1 exhibited the highest enzymatic activity at pH 8, which is equivalent to the optimum pH of free rlevblg1 [39]. However, above pH 8, the attained activity of rlevblg1-OdexM-CLEAs was higher than that of the free enzyme. At pH 11, immobilized rlevblg1 retained 5.8-fold higher catalytic activity than the free enzyme. Thus, after cross-linking process, the newly formed linkages between the enzyme molecules and cross-linker (OdexM) has influenced the structural conformation and altered the microenvironment surrounding the active sit of the enzyme [75], that of which are responsible for the catalytic activity and sensitivity to pH [76]. This trend is consistent with the findings of Cui et al. [19], where phenylalanine ammonia lyase-M-CLEAs exhibited higher relative activity in a wide pH range of 8–10 compared to its free form.

The pH stability of rlevblg1-OdexM-CLEAs was evaluated after incubation for 1 h with the absence of the substrate at different pH values ranging from pH 6–11. Based on Fig 8 (B), rlevblg1-OdexM-CLEAs was most stable at the slightly alkaline condition, pH 8. Moreover, at acidic (pH 6), natural (pH 7) and alkaline (pH 9–10) environments, rlevblg1-OdexM-CLEAs maintained excellent stability with the retain of more than 80% of its initial activity. Additionally, at pH 11, rlevblg1-OdexM-CLEAs retained 60.7% of its relative activity. In contrast, laccase-M-CLEAs [77] and M-CLEAs combining horseradish peroxidase and glucose oxidase [78] exhibited a narrower pH tolerance, with a decline in the activity outside their optimal pH range.

Furthermore, rlevblg1-OdexM-CLEAs displayed greater stability in acidic and alkaline environments compared to the free enzyme and rlevblg1-dex-CLEAs [39]. At pH 6, M-CLEAs of rlevblg1 exhibited 1.1-fold higher pH stability than that of free enzyme and CLEAs. At extremely alkaline conditions (pH 11), rlevblg1-OdexM-CLEAs exhibited 2 and 3.4-fold greater pH resistance than free and CLEAs of rlevblg1, respectively. The increase in tolerance to variations of pH is due to the formation of strong covalent interactions between OdexM and rlevblg1 [18,79], which altered the configuration of the enzyme, thus improving its relative activity in a wide range of pH adaptability [76,80].

#### Optimum temperature and thermal stability of rlevblg1-OdexM-CLEAs.

**Fig 9:**
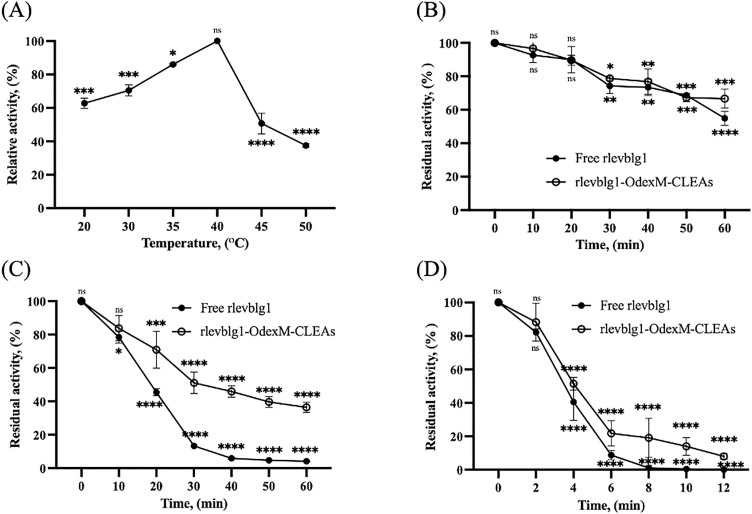
Optimum temperature and thermal stability. (A) Optimum temperature of rlevblg1-OdexM-CLEAs. The optimum temperature was adjusted as 100%. Thermal stability of free and M-CLEAs of rlevblg1 at (B) 35 °C, (C) 40 °C, and (D) 45 °C. The initial activity was adjusted as 100%. The experiments were performed in triplicate, and error bars indicate standard deviations. Average values that show significant differences are highlighted with varying numbers of asterisks. This is based on the results from a one-way ANOVA followed by Tukey’s multiple comparisons test, using a 95% confidence interval: p < 0.05 (*) indicates significance; p < 0.01 (**) indicates a more significant difference; p < 0.001 (***) denotes high significance; p < 0.0001 (****) indicates an extremely significant difference, while (ns) signifies a non-significant result.

The optimum temperature of rlevblg1-OdexM-CLEAs was observed by measuring the enzymatic activity at particular temperatures ranging from 20 °C to 50 °C. As shown in Fig 9 (A), the optimum temperature of rlevblg1-OdexM-CLEAs was 40 °C to reach the maximum activity. Interestingly, rlevblg1 cross-linked with OdexM represented a higher optimal temperature than that cross-linked with dialdehyde starch (DAS) (30 °C) [53] and native dextran (35°C) [39]. This could be due to the occurrence of different intermolecular interactions between the enzyme molecules and each cross-linker, which led to the varying conformational change of the immobilized rlevblg1, thus altering the required temperature for the initiation of the catalytic reaction [81]. Accordingly, OdexM has formed strong bonding with the enzyme molecules, hence possessing higher temperature resistance [79]. Therefore, the optimum temperature of rlevblg1-OdexM-CLEAs has shifted from 30 °C of the free enzyme to 40 °C, which is consistent with the study by Feng et al. [76], where the optimum temperature of immobilized aminoacylase with MNPs is 10 °C higher than that of the free enzyme.

The thermal stability of free rlevblg1 and M-CLEAs was evaluated after incubation at various temperatures (35 °C, 40 °C and 45 °C) for different time intervals (Fig 9 (B-D)). After 1 h of incubation at 35 °C, rlevblg1-OdexM-CLEAs retained almost 70% of its initial activity. At 40 °C, the residual activity decreased to 51.1% and 36.4% after 30 min and 1 h of incubation, respectively.

Compared to free rlevblg1, M-CLEAs exhibited a significant improvement in the thermal stability. After 30 min and 1 h of incubation at 40 °C, the residual activity of rlevblg1-OdexM-CLEAs was 3.9 and 8.9-fold higher than that of the free enzyme, respectively. In addition, at 45 °C, rlevblg1-OdexM-CLEAs retained almost 20% of its initial activity, whereas all of the free enzyme activity was lost after 8 min of incubation. The improvement in the thermal stability after immobilization is due to the formed intermolecular interactions between the enzyme molecules and cross-linker [82] that provided additional structural stabilization to the enzyme [83], and protected its conformation from heat distortion [79]. Several studies have reported that M-CLEAs of different enzymes such as acylamidase [32], l-arabinose isomerase [84], lipase [85], laccase [77,86], and cellulase [28] had higher thermal stability than their free enzymes. The ability of M-CLEA to withstand and function under elevated temperatures enhances the bioprocess efficiency and allows its use as a biocatalyst in various industrial applications.

#### Kinetic and effectiveness factor analyses.

The kinetic parameters of enzymes, including *K*_*m*_, V_max_, k_cat_, and k_cat_/*K*_*m*_, provide important information about the catalytic efficiency and evaluate the performance of the biocatalyst [87]. Therefore, the kinetic analysis of rlevblg1-OdexM-CLEAs was measured using Lineweaver Burk plot. The resulting kinetic parameters were listed in S1 Table and compared to the performance of the free and CLEAs forms of rlevblg1 reported in our previous study [39]. The V_max_ value of M-CLEAs (4.46 mM.min^−1^) was lower than that of rlevblg1-dex-CLEAs (8.38 mM.min^−1^) and free enzyme (10.26 mM.min^−1^). The turnover number (k_cat_) of rlevblg1-OdexM-CLEAs (5.16 s^−1^) was also lower than that of rlevblg1-dex-CLEAs (9.70 s^−1^) and free enzyme (11.88 s^−1^). Despite M-CLEAs having a reduced value of V_max_ and k_cat_, influenced by its 74.7% activity retention, rlevblg1-OdexM-CLEAs demonstrated a stronger affinity for the substrate, indicated by a lower *K*_*m*_ value. The *K*_*m*_ value of rlevblg1-OdexM-CLEAs (7.32 mM) was significantly lower than that of CLEAs (13.79 mM) and the free enzyme (14.80 mM). The decrease in the *K*_*m*_ value of M-CLEAs confirms a higher affinity toward the substrate, which is presumably due to the changes in the enzyme conformation after the aggregation and cross-linking processes [88]. Compared to CLEAs of rlevblg1, M-CLEAs exhibited a further increase in the substrate affinity, indicating that the use of OdexM provided a more suitable conformational and micro-environmental alteration [75], which improved the interaction between the immobilized enzyme and substrate. Thus, rlevblg1-OdexM-CLEAs exhibited an enhancement in the substrate accessibility and displayed an excellent catalytic efficiency, which is better than that of free enzyme, considering its activity recovery.

Furthermore, the study of the effectiveness factor is particularly important when using cross-linked enzyme aggregates (CLEAs) or magnetic-CLEAs, which provides insights into the mass transport limitations that affect the immobilized enzyme activity and performance. Therefore, the effectiveness factor (ƞ) of rlevblg1-OdexM-CLEAs was investigated by applying different concentrations of levan ranging from 1.0 to 25.0 mg/mL. As demonstrated in Table 1, the effectiveness factor was low at minimal concentration of levan (1, 2, and 4 mg/mL), which could be due to internal mass transfer limitations or slow diffusion of the substrate into the active site of the immobilized enzyme [87]. With a higher than 4 mg/mL concentration of levan, the effectiveness factor drastically increased and was equivalent to that of the free enzyme. Thus, the diffusion of the levan into the active site of rlevblg1-OdexM-CLEAs has been accelerated with increasing substrate concentration.

**Table 1:** Effectiveness factors (ƞ) of rlevblg1-OdexM-CLEAs at different levan concentrations.

Levan mg/mL	Velocity (μmole/mg.min)	ƞ
1	0.14 ± 0.10	0.29
2	0.95 ± 0.45	0.67
4	1.51 ± 0.71	0.53
6	2.97 ± 0.27	0.99
8	3.20 ± 0.38	0.94
10	4.05 ± 0.34	0.92
12	4.66 ± 0.04	1.00
14	4.69 ± 0.00	0.97
20	4.81 ± 0.06	0.97
25	3.50 ± 0.18	0.90

Moreover, rlevblg1-OdexM-CLEAs had a higher effectiveness factor, especially at high levan concentrations (6.0–25.0 mg/mL) compared to rlevblg1-dex-CLEAs [39]. This is assumed to be due to the added benefit of OdexM, which provides a higher surface-to-volume ratio that increases immobilization efficiency as well as reduces diffusional and mass transfer limitations [76,89]. Therefore, rlevblg1-OdexM-CLEAs had a lower *K*_*m*_ value than that of rlevblg1-dex-CLEAs which indicates a higher binding towards the substrate, thus increasing substrate accessibility and improving catalytic efficiency, as a result highlighting its potential for L-FOS industrial production.

#### Mechanical stability.

Mechanical analysis is essential to measure the maintaining catalytic activity and stability of the immobilized enzyme. The mechanical stability of M-CLEAs particles is mainly affected by the efficiency of the cross-linking process between the enzyme molecules and the cross-linking agent [90,91]. Mechanically unstable M-CLEAs particles would cause enzyme leaching from the aggregates, resulting in the reduction of the recovered activity and reusability of the immobilized enzyme [22]. Therefore, in this study, computational analysis was used to improve the efficiency of cross-linking by predicting the intermolecular interactions between the enzyme and cross-linker (Fig 2), thus enhancing the mechanical stability.

**Fig 10:**
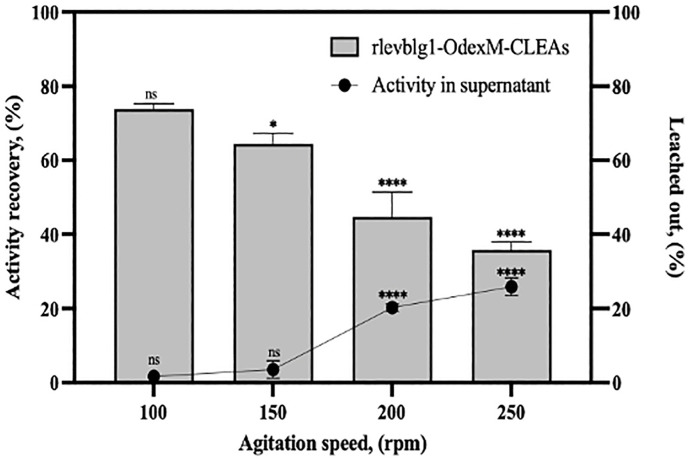
Mechanical and leaching analyses of rlevblg1-OdexM-CLEAs after shaking at various agitation speeds for 30 min at 30 °C. The experiment was conducted in triplicate and error bars represent standard deviations. Average values that show significant differences are highlighted with varying numbers of asterisks. This is based on the results from a one-way ANOVA followed by Tukey’s multiple comparisons test, using a 95% confidence interval: p < 0.05 (*); p < 0.0001 (****) indicates an extremely significant difference, while (ns) signifies a non-significant result.

The mechanical and leaching analyses of rlevblg1-OdexM-CLEAs were investigated using different agitation speeds (100, 150, 200 and 250 rpm) to determine its stability and suitability for industrial applications. As illustrated in Fig 10, at 100 rpm, rlevblg1-OdexM-CLEAs was remarkably stable and retained its full recovery activity (74%) at the optimal condition (Fig 4), therefore, there was no sign of any enzymatic activity in the supernatant. At 150 rpm, a slight reduction in the activity recovery with minimal leakage of the aggregates were detected. In addition, more than 40% of the initial activity and 20% of leaching were observed at 200 rpm. At 250 rpm, about half (35%) of the full activity was recovered and 25.8% of rlevblg1 has leached into the reaction mixture. The excellent mechanical resistance of rlevblg1-OdexM-CLEAs is attributed to the strong bonding between rlevblg1 and OdexM as well as to the applications of an external magnetic field for the separation process [21].

Separation of the immobilized biocatalyst via centrifugation increases the compactness of CLEAs and compromises its mechanical robustness leading to enzyme leaching and loss of the enzymatic activity [28,92]. On the other hand, magnetic separation allows easy separation and reduces the risk of mechanical stress on the aggregates; therefore M-CLEAs exhibits better mechanical stability than CLEAs and similar catalytic performance to that of the free enzyme [21,28]. Herein, M-CLEAs of rlevblg1 demonstrated a greater mechanical stability and less leaching out at all agitation speeds compared to rlevblg1-CLEAs (rlevblg1 cross-linked with dialdehyde starch (DAS)) [53]. Cross-linking rlevblg1 with DAS retained up to 50% of its initial activity with almost 40% leaching of the enzyme at 150 rpm. By increasing the agitation speed to 250 rpm, less than 20% of the enzyme was recovered while 45% leached out. Beside the effect of centrifugation on the mechanical stability, the continuous decrease in the recovered activity and high percentage of enzyme leaching might be due to the inefficient cross-linking and weak binding affinity (−4.7 kcal/mol) between rlevblg1 and DAS, which was predicted earlier through molecular docking [27].

#### Reusability.

**Fig 11:**
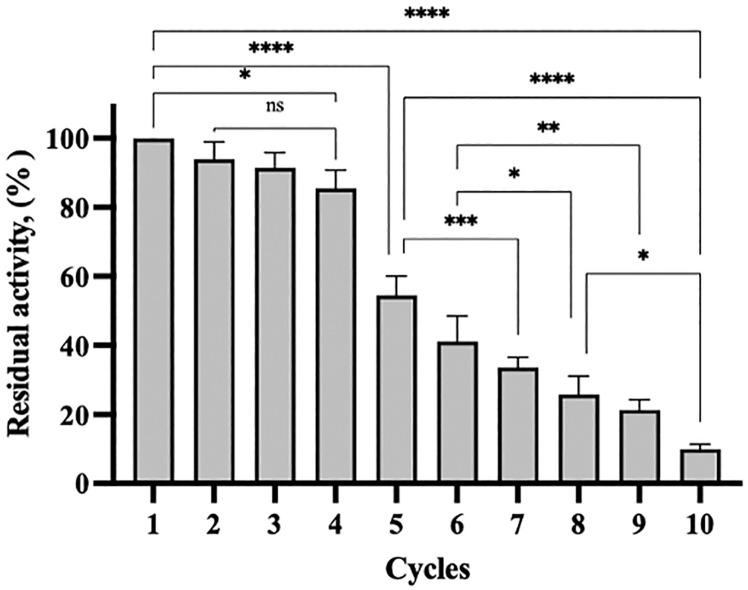
Reusability of rlevblg1-OdexM-CLEAs for ten cycles. The initial activity was adjusted as 100%. The experiments were performed in triplicate, and error bars indicate standard deviations. Average values that show significant differences are highlighted with varying numbers of asterisks. This is based on the results from a one-way ANOVA followed by Tukey’s multiple comparisons test, using a 95% confidence interval: p < 0.05 (*) indicates significance; p < 0.01 (**) indicates a more significant difference; p < 0.001 (***) denotes high significance; p < 0.0001 (****) indicates an extremely significant difference, while (ns) signifies a non-significant result.

Reusability of immobilized enzymes is a substantial characteristic due to the provided economic viability and practical application in industrial bioprocesses [83]. M-CLEAs displayed the potential in developing robust, easily recovered and recyclable biocatalysts that could be used in many industrial applications [21,31]. As presented in Fig 11, rlevblg1-OdexM-CLEAs was reused for ten cycles and retained similar activity as its initial activity for four consecutive cycles. Using an immobilized enzyme that maintains its activity and selectivity for several cycles is exemplary for industrial applications [80]. At the fifth cycle, the retained activity was almost 60% and continued to gradually reduce as the number of the cycles increased. After ten cycles of reuse, the remaining activity was between 10.5–11%. The decrease in the activity after multiple cycles of use might be attributed to enzyme leaching during the washing at the end of each cycle [93]. Moreover, the solution environment presumably caused changes in the integrity of the enzyme conformation, and OdexM was dismantled from rlevblg1 molecules resulting in the reduction in the activity [76].

Nevertheless, using OdexM to cross-link rlevblg1 has expanded the cycle numbers of reuse to ten cycles compared to that of cross-linked with dextran (5 cycles) [39] and DAS (7 cycles) [53]. Moreover, M-CLEAs of rlevblg1 retained better residual activity than that of CLEAs. Concluding the fifth cycle, M-CLEAs of rlevblg1 retained 5.4- and 1.5-fold higher residual activity than that of CLEAs formed using dextran and DAS as cross-linkers, respectively. In addition, rlevblg1-OdexM-CLEAs retained 34%, whereas less than 20% of the initial activity of rlevblg1-DAS-CLEAs was obtained after the seventh cycle. The increase in the cycle numbers and retained activity observed in M-CLEAs is due to the formation of stronger intermolecular interactions between the enzyme molecules and OdexM than those formed between rlevblg1 and dextran or DAS. In addition, the replacement of centrifugation with a magnetic force during the separation process of rlevblg1-OdexM-CLEAs would increase the cycle numbers of reuse; excessive or repeated centrifugation may cause structural damage to CLEAs particles, potentially weakening the bonding and reducing the reusability [64]. With the features of magnetic separation and enhanced reusability, endolevanase-M-CLEAs is particularly valuable in continuous bioprocessing and large-scale productions of L-FOS, where enzyme cost and recovery are major concerns.

### L-FOS production

The hydrolysis of levan via M-CLEAs of rlevblg1 for L-FOS was analyzed under the standard condition (30 °C, pH 8, 850 × g) at different reaction times (1, 2 and 3 h) (Table 2). At all reaction times, rlevblg1-OdexM-CLEAs mostly hydrolyzed levan to levanpentaose (DP5) followed by levanbiose (DP2) and levantriose (DP3). Notably, fructose and levantetraose (DP4) were not present among the final products even after extending the reaction time to 3 h, indicating the high specificity and selectivity of the developed biocatalyst in hydrolyzing levan [8].

**Table 2:** L-FOS synthesis through the hydrolysis of 0.5% (w/v) of soluble levan by rlevblg1-OdexM-CLEAs at 30 °C for 1, 2, and 3 h of reaction time.

Time (h)	Fructose (mg/g)	DP2 (mg/g)	DP3 (mg/g)	DP5 (mg/g)	Total reducing sugar (mg/g)	Total L-FOS (mg/g)	Product ratio
1	0.0 ± 0.00	150.2 ± 0.21	49.3 ± 5.75	1450.8 ± 1.56	1650.5	1650.5	1.0
2	0.0 ± 0.00	286.9 ± 13.10	39.1 ± 1.73	1465.5 ± 1.07	1791. 6	1791. 6	1.0
3	0.0 ± 0.00	257.2 ± 4.83	120.8 ± 16.45	1602.1 ± 0.08	1980.2	1980.2	1.0

Based on previous studies, using different cross-linkers to immobilize rlevblg1 influenced the hydrolysis reaction of levan, resulting in the alteration of the main product of L-FOS. Cross-linking rlevblg1 with glutaraldehyde [5] and DAS [53] produced DP2 and DP3 as their main product, respectively. Interestingly, rlevblg1 cross-linked with dextran [39] and OdexM retained the same main product (DP5) of L-FOS. The production of L-FOS with higher DP is attributed to the strong binding between the cross-linker (dextran/ OdexM) and rlevblg1 which formed porous structures, hence facilitating substrate accessibility, affecting water activity and increasing hydrophobicity at the active site, potentially leading to the reduction in the hydrolysis and the formation of longer L-FOS.

Nevertheless, compared to rlevblg1-dex-CLEAs [39], the total percentage of levan bioconversion by M-CLEAs of rlevblg1 was 1-fold higher at all reaction times. Furthermore, the structure of rlevblg1-OdexM-CLEAs was more porous (as confirmed by the particle size distribution); hence mass transfer limitation was decreased, substrate accessibility was increased and catalytic properties were improved, which was confirmed by the lower *K*_*m*_ and higher effectiveness factor values. Importantly, M-CLEAs exhibited significantly better thermal stability than that of CLEAs, thus it resisted the high temperature of the reaction, slowed the hydrolysis rate and prevented any re-hydrolysis activity for the final product. Therefore, rlevblg1-OdexM-CLEAs hydrolyzed levan to only L-FOS, whereas fructose was detected in the final product of rlevblg1-dex-CLEAs after 2 and 3 h of reaction.

In addition, compared to other L-FOS producing enzymes, including levansucrase and LFTase, M-CLEAs of rlevblg1 is a superior biocatalyst for the efficient, high-purity and scalable production of L-FOS. Levansucrase produces an inconsistent L-FOS profile with glucose as a by-product [3], which can inhibit further enzyme activity and complicate downstream processing. On the other hand, LFTase catalyzes the conversion of levan into a modified L-FOS compound (difructose anhydride IV (DFA IV)) [4], potentially altering their functional properties. Herein, M-CLEAs of endolevanase exhibits specificity in generating monosaccharide-free L-FOS during levan hydrolysis, which is crucial for tailoring prebiotic functionalities. Thus, rlevblg1-M-CLEAs provides a novel combination of easily recovered biocatalysts that can be reused in multiple catalytic cycles to produce well-defined L-FOS profiles efficiently, with no by-products and an enhanced suitability for industrial applications.

## Conclusion

The immobilization of rlevblg1 into magnetic cross-linked enzyme aggregates (M-CLEAs) was successfully achieved using a combination of computational aids to avoid undesired bonding and produce L-FOS. Compared to acetyl -dextran, oxidized dextran (Odex) displayed distanced interactions from the active site of the enzyme. Therefore, it was used to cover the magnetic nanoparticles (MNPs) and develop OdexM, which was applied as a cross-linking agent. The formed rlevblg1-OdexM-CLEAs was magnetically recovered with a high recovery rate of over 74.7%. In terms of operational properties, M-CLEAs had the same optimum pH and kinetic performance as the free and CLEA forms of rlevblg1. However, M-CLEAs exhibited a higher optimum temperature and improved the thermal and pH stability, which ensure sustained catalytic activity under the harsh operational conditions commonly encountered in industrial processes. Moreover, rlevblg1-OdexM-CLEAs obtained an enhanced mechanical stability and reusability, highlighting their potential as promising biocatalysts for applications in the food industry, particularly for large-scale L-FOS productions.

## Supporting information

S1 Fig HPLC standard curve for fructose. (DOCX)

S2 Fig HPLC standard curve for glucose. (DOCX)

S3 Fig HPLC standard curve for levanbiose (DP2). (DOCX)

S4 Fig HPLC standard curve for levantriose (DP3). (DOCX)

S5 Fig HPLC standard curve for levantetraose (DP4). (DOCX)

S6 Fig HPLC standard curve for levanpentaose (DP5). (DOCX)

S1 Table Kinetic parameters of rlevblg1-OdexM-CLEAs. (DOCX)

S1 Data Raw Data. (DOCX)
