# Supplementary figures and images for: Oxidized dextran coated magnetic nanoparticles to develop magnetic cross-linked Bacillus lehensis G1 endolevanase aggregates for levan-type fructooligosaccharides synthesis
Source: PLoS One. 2025 Oct 15;20(10):e0333803. doi: 10.1371/journal.pone.0333803 (PMC12527180; doi:10.1371/journal.pone.0333803)

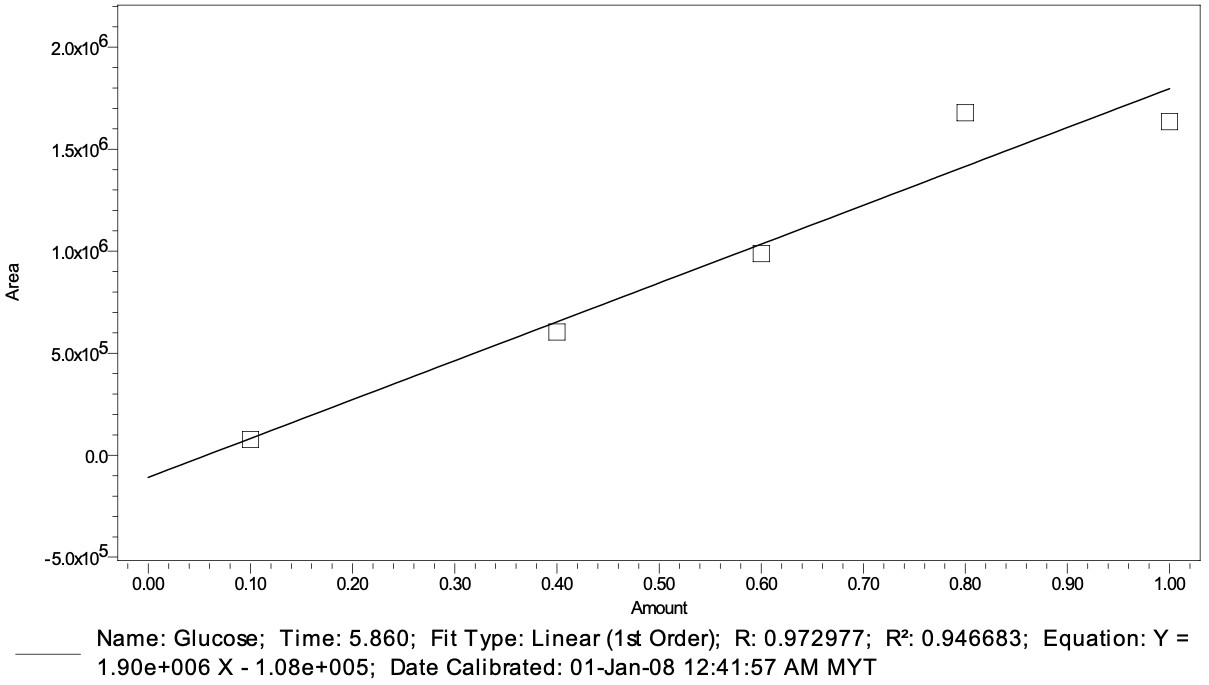


**S2 Fig.** HPLC standard curve for glucose

Supplement: S1 Fig — (DOCX) [file pone.0333803.s001.docx]

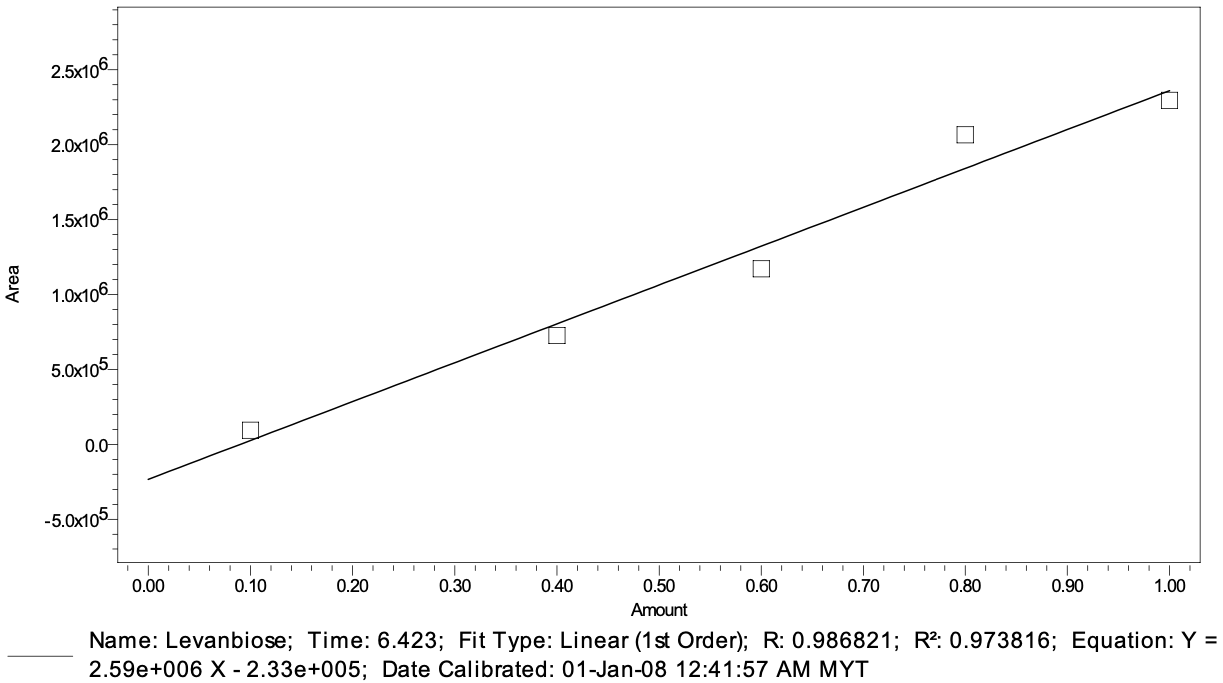


**S3 Fig.** HPLC standard curve for levanbiose (DP2)

Supplement: S3 Fig — (DOCX) [file pone.0333803.s003.docx]

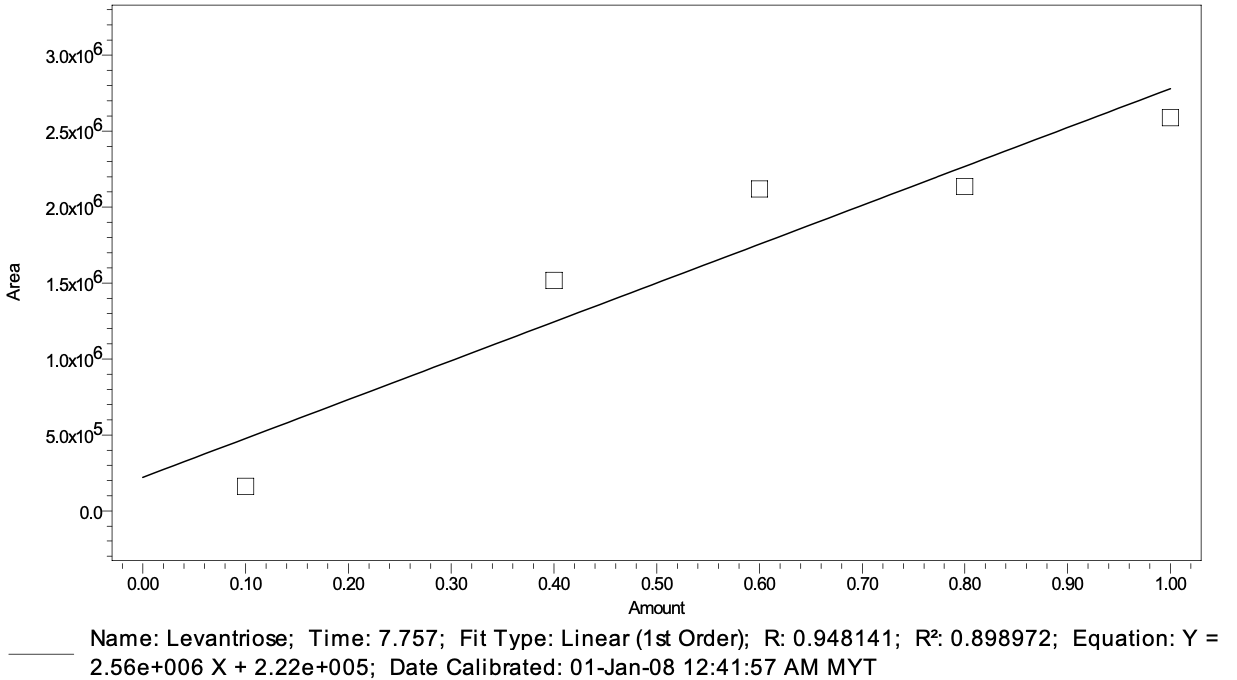


**S4 Fig.** HPLC standard curve for levantriose (DP3)

Supplement: S4 Fig — (DOCX) [file pone.0333803.s004.docx]

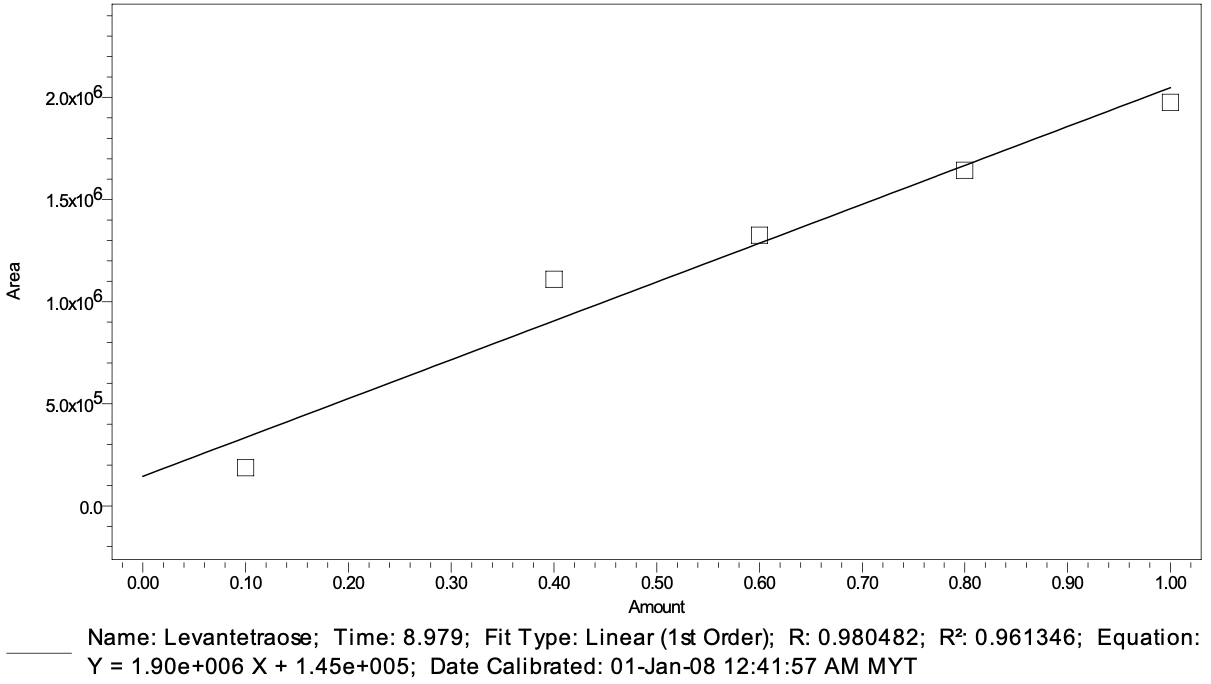


**S5 Fig.** HPLC standard curve for levantetraose (DP4)

Supplement: S5 Fig — (DOCX) [file pone.0333803.s005.docx]

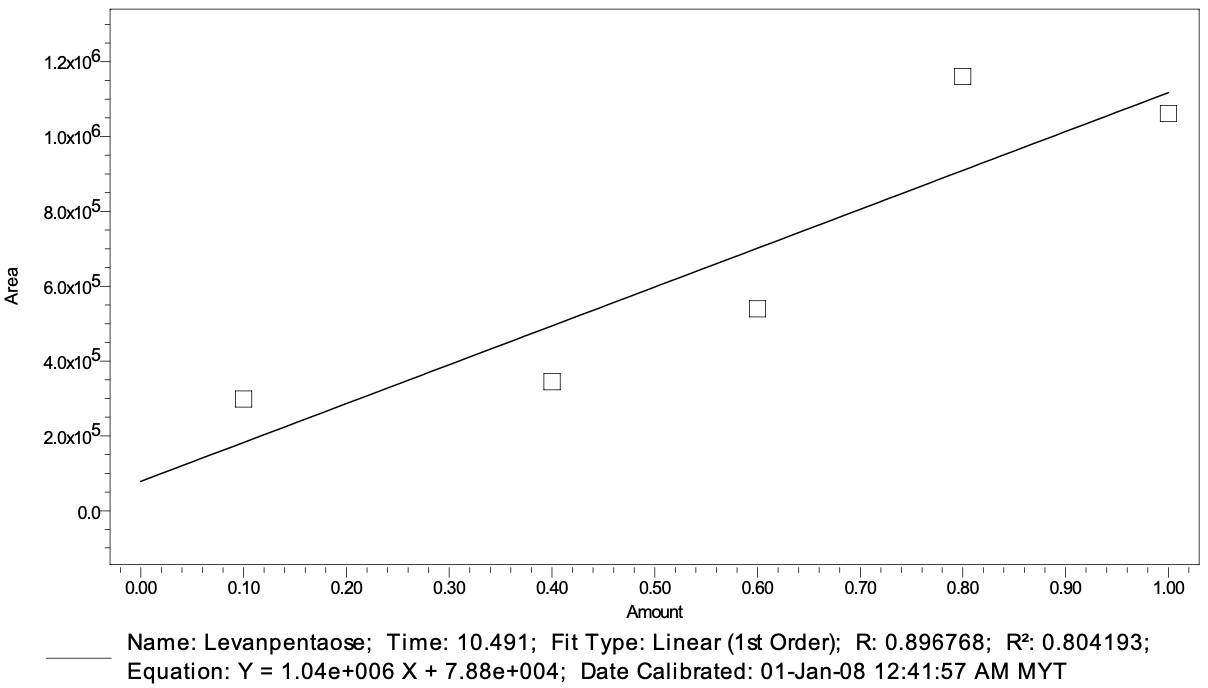


**S6 Fig.** HPLC standard curve for levanpentaose (DP5)

Supplement: S6 Fig — (DOCX) [file pone.0333803.s006.docx]
