# Supplementary material for: Oxidized dextran coated magnetic nanoparticles to develop magnetic cross-linked Bacillus lehensis G1 endolevanase aggregates for levan-type fructooligosaccharides synthesis
Source: PLoS One. 2025 Oct 15;20(10):e0333803. doi: 10.1371/journal.pone.0333803 (PMC12527180; doi:10.1371/journal.pone.0333803)
Supplement: S1 Table — (DOCX) [file pone.0333803.s007.docx]

**S1 Table.** Kinetic parameters of rlevblg1-OdexM-CLEAs

| V_max_ (mM.min^−1^) | *K_m_* (mM) | k_cat_ (s^−1^) | Catalytic efficiency (k_cat_/*K_m_*) (mM^−1^s^−1^) |
| --- | --- | --- | --- |
| 4.46 ± 2.4 | 7.32 ± 0.5 | 5.16 ± 2.7 | 0.73 ± 0.4 |
